# Subtypes of Familial Hemophagocytic Lymphohistiocytosis in Japan Based on Genetic and Functional Analyses of Cytotoxic T Lymphocytes

**DOI:** 10.1371/journal.pone.0014173

**Published:** 2010-11-30

**Authors:** Kozo Nagai, Ken Yamamoto, Hiroshi Fujiwara, Jun An, Toshiki Ochi, Koichiro Suemori, Takahiro Yasumi, Hisamichi Tauchi, Katsuyoshi Koh, Maho Sato, Akira Morimoto, Toshio Heike, Eiichi Ishii, Masaki Yasukawa

**Affiliations:** 1 Department of Bioregulatory Medicine, Ehime University Graduate School of Medicine, Ehime, Japan; 2 Department of Pediatrics, Ehime University Graduate School of Medicine, Ehime, Japan; 3 Department of Molecular Genetics, Medical Institute of Bioregulation, Kyushu University, Fukuoka, Japan; 4 Proteo-Medicine Research Center, Ehime University, Ehime, Japan; 5 Department of Pediatrics, Graduate School of Medicine, Kyoto University, Kyoto, Japan; 6 Department of Pediatrics, Graduate School of Medicine, University of Tokyo, Tokyo, Japan; 7 Department of Pediatrics, Osaka Medical Center and Research Institute for Maternal and Child Health, Osaka, Japan; 8 Department of Pediatrics, School of Medicine, Jichi Medical University, Tochigi, Japan; INSERM U768, Pavillon Kirmisson, France

## Abstract

**Background:**

Familial hemophagocytic lymphohistiocytosis (FHL) is a rare disease of infancy or early childhood. To clarify the incidence and subtypes of FHL in Japan, we performed genetic and functional analyses of cytotoxic T lymphocytes (CTLs) in Japanese patients with FHL.

**Design and Methods:**

Among the Japanese children with hemophagocytic lymphohistiocytosis (HLH) registered at our laboratory, those with more than one of the following findings were eligible for study entry under a diagnosis of FHL: positive for known genetic mutations, a family history of HLH, and impaired CTL-mediated cytotoxicity. Mutations of the newly identified causative gene for FHL5, *STXBP2*, and the cytotoxicity and degranulation activity of CTLs in FHL patients, were analyzed.

**Results:**

Among 31 FHL patients who satisfied the above criteria, *PRF1* mutation was detected in 17 (FHL2) and *UNC13D* mutation was in 10 (FHL3). In 2 other patients, 3 novel mutations of *STXBP2* gene were confirmed (FHL5). Finally, the remaining 2 were classified as having FHL with unknown genetic mutations. In all FHL patients, CTL-mediated cytotoxicity was low or deficient, and degranulation activity was also low or absent except FHL2 patients. In 2 patients with unknown genetic mutations, the cytotoxicity and degranulation activity of CTLs appeared to be deficient in one patient and moderately impaired in the other.

**Conclusions:**

FHL can be diagnosed and classified on the basis of CTL-mediated cytotoxicity, degranulation activity, and genetic analysis. Based on the data obtained from functional analysis of CTLs, other unknown gene(s) responsible for FHL remain to be identified.

## Introduction

Hemophagocytic lymphohistiocytosis (HLH) is characterized by fever and hepatosplenomegaly associated with pancytopenia [1]–[3]. Histologically, infiltration of lymphocytes and histiocytes with hemophagocytic activity is evident in the reticuloendothelial system, bone marrow, and central nervous system [4]. HLH can be classified as either primary or secondary [5]. Primary HLH, also known as familial hemophagocytic lymphohistiocytosis (FHL), is inherited as an autosomal recessive disorder that usually arises during infancy.

The pathogenesis of FHL has been considered to involve dysfunction of cytotoxic T lymphocyte (CTL) activity, leading to excessive production of inflammatory cytokines and macrophage activation [6]. The genetic mutations responsible for FHL have been identified by various methods. Linkage analysis has indicated two possible loci: FHL1 (MIM 603552) in 9q21.3-22, and FHL2 (MIM 603553) in 10q21-22 [7], [8]. In 1999, a mutation in the *perforin* gene (*PRF1*) was identified as the cause of FHL2 [9]–[12]. Further genetic mutations of the *Munc13-4* gene (*UNC13D*) mapped to 17q25 (the cause of FHL3, MIM 608898) and the *syntaxin11* gene (*STX11*) mapped to 6q24 (the cause of FHL4, MIM 603552) were subsequently identified [13]–[15]. These mutations affect proteins involved in the transport and membrane fusion, or exocytosis, of perforin contained in cytoplasmic granules. Recently, mutations of the *Munc18-2* gene (*STXBP2*), located in 19q, were detected as a cause of FHL5 [16], [17]. Munc18-2 regulates intracellular trafficking and controls the soluble N-ethylmaleimide-sensitive fusion factor attachment protein receptor (SNARE) complex.

The molecular mechanisms underlying vesicular membrane trafficking and regulation of exocytosis have been clarified in recent years. The final step of vesicle transport is mediated by a bridge between a vesicle and its target membrane through formation of a ternary complex between a vesicle-SNARE (v-SNARE), such as a VAMP, and a target membrane-SNARE (t-SNARE), such as a syntaxin11 or a member of SNAP23/25/29 [18]. The SNARE complex is composed of three molecules: VAMP, syntaxin and SNAP23/25/29. Syntaxin11, in association with SNAP23, localizes to the endosome and trans-Golgi network [19]; however, the precise biological functions of the SNARE system are still poorly understood. Recent evidence suggests that members of the SNARE family mediate fusion of cytotoxic granules with the surface of CTLs. Syntaxin11, SNAP23 and VAMP7 are prime candidates for functioning as SNAREs in this fusion event [20].

It has been considered that clarification of the molecular abnormalities in FHL might shed light on the mechanisms of CTL-mediated cytotoxicity. Accordingly, we have been studying the functional abnormalities of CTLs in Japanese patients with FHL [21]. Our previous studies have shown that the FHL2 and FHL3 subtypes account for 20–25% of all FHL cases, respectively, whereas no FHL4 subtype exists; therefore, 45–50% of FHL cases in Japan harbor still unknown genetic mutations [21], [22]. However, secondary HLH could be involved in patients with unknown genetic mutations, because both FHL and secondary HLH share similar clinical and laboratory characteristics. Therefore, in the present study aimed at clarifying the incidence and subtypes of FHL in Japanese children by genetic and functional analyses of CTLs, only patients positive for known genetic mutations, a positive family history of HLH, or impaired natural killer (NK)/CTL-mediated cytotoxicity were diagnosed definitively as having FHL.

## Materials and Methods

### Patients

A total of 87 Japanese children aged <15 years diagnosed as having HLH based on the diagnostic criteria of the Histiocyte Society [23] were registered at our laboratory between January 1994 and December 2009. Among them, 40 were excluded from analysis because they were diagnosed as having secondary HLH, or their parents did not provide permission for use of clinical samples. None of the patients had Chediak-Higashi syndrome, Griscelli syndrome, or Hermansky-Pudlak syndrome type 2, based on clinical and laboratory findings, including albinism or the presence of gigantic granules in lymphocytes or granulocytes. A final total of 31 patients, who met the diagnostic criteria for FHL, and for whom documented informed consent had been obtained in accordance with the Declaration of Helsinki, were entered into the study.

### Genetic analysis of the *STXBP2* gene

For the detection of *STXBP2* mutations, genomic DNA was isolated from a T-cell line established from each patient. Genomic DNA (5 ng) was subjected to PCR using the primers listed in Table S1. These primer sets were designed to amplify 19 exons including the 5′-untranslated region and the coding regions with the exon-intron boundaries of *STXBP2*. The PCR products were treated with ExoSAP-IT (GE Healthcare Bio-Sciences, Little Chalfont, England) by incubation at 37°C for 15 minutes to inactivate the free primers and dNTPs, and then subjected to sequencing reactions using forward or reverse primers and BigDye® Terminator v3.1 (Applied Biosystems, Foster City, CA). The DNA fragments were purified using Magnesil (Promega, Madison, WI), and sequencing was carried out with an ABI 3730 Genetic Analyzer (Applied Biosystems). Sample sequences were aligned to reference sequences obtained from the UCSC Genome Bioinformatics website (http://genome.ucsc.edu/index.html) using the ClustalW program in order to identify nucleotide changes. Mutations were numbered according to GenBank Reference Sequence NM_001127396.1; additionally, the A of the ATG initiator codon was defined as nucleotide +1. To identify splicing variants generated by c.88-1g>a mutation of *STXBP2*, total RNA was extracted from each patient's T-cell line and reverse transcriptase PCR (RT-PCR) was performed using the forward primer on exon 1 (5′-TTGGGACACACCCGGAAG-3′) and the reverse primer on exon 5 (5′-AAGAAGATATGGGCCGCTTT-3′). The PCR products were directly sequenced using the forward primer, as described above.

### Western blot analysis of MUNC18-2 protein

Expression of Munc18-2 protein encoded by *STXBP2* in T-cell lines established from FHL patients and a healthy individual was analyzed by Western blotting. CTLs were harvested after 5 days of stimulation with allogeneic LCL cells. Cell lysates were then prepared by extraction with 1% NP-40, and the extracts (10 µg per lane) were analyzed by Western blotting with anti-Munc18-2 rabbit polyclonal antibody (LifeSpan BioSciences, Seattle, WA). Horseradish peroxidase-labeled anti-rabbit IgG polyclonal antibody was used as the secondary antibody with detection by enhanced chemiluminescence (Amersham Biosciences, Buckinghamshire, UK).

### Establishment of alloantigen-specific CTL lines

Alloantigen-specific CD8^+^ CTL lines were generated as described previously [24], [25]. Briefly, peripheral blood mononuclear cells (PBMCs) were obtained from FHL patients and unrelated healthy individuals. These cells were co-cultured with a mitomycin C (MMC)-treated B-lymphoblastoid cell line (B-LCL) established from an HLA-mismatched individual (KI-LCL). Using cell-isolation immunomagnetic beads (MACS beads) (Miltenyi Biotec, Auburn, CA), CD8^+^ T lymphocytes were isolated from PBMCs that had been stimulated with KI-LCL cells for 6 days. CD8^+^ T lymphocytes, cultured in RPMI 1640 medium supplemented with 10% human serum and 10 IU/ml interleukin-2 (Roche, Mannheim, Germany), were stimulated with MMC-treated KI-LCL cells 3 times at 1-week intervals; subsequently, these lymphocytes were used as CD8^+^ alloantigen-specific CTL lines. The alloantigen specificity of the CTL lines was determined by assay of interferon-γ (IFN-γ) production in response to stimulation with KI-LCL cells, as described previously [24], [25]. Briefly, 1×10^5^ T lymphocytes were co-cultured with or without 1×10^5^ MMC-treated B-LCL cells in 0.2 ml of RPMI 1640 medium supplemented with 10% fetal calf serum (FCS) in a flat-bottomed 96-well plate. In some experiments, an anti-HLA class I monoclonal antibody (w6/32; American Type Culture Collection, Manassas, VA) was added to wells at an optimal concentration. After 24 hours, the supernatant was collected from each well and assayed for production of IFN-γ using an enzyme-linked immunosorbent assay (ELISA; ENDOGEN, Woburn, MA).

### Analysis of CTL-mediated cytotoxicity

The cytotoxic activity of CTLs was measured by a standard ^51^Cr-release assay, as described previously [21]. Briefly, alloantigen-specific CTLs were incubated with ^51^Cr-labeled allogeneic KI-LCL cells or TA-LCL cells for 5 hours at an effector:target cell ratio (E/T) of 2.5∶1, 5∶1, and 10∶1. Target cells were also added to wells containing medium alone and to wells containing 0.2% Triton X-100 to determine the spontaneous and maximal levels of ^51^Cr release, respectively. After 5 hours, 0.1 ml of supernatant was collected from each well. The percentage of specific ^51^Cr release was calculated as (cpm experimental release - cpm spontaneous release)/(cpm maximal release - cpm spontaneous release) ×100, where cpm indicates counts per minute.

### Degranulation analysis by flow cytometry

Degranulation activity was analyzed by flow cytometry using anti-CD107a antibody (BioLegend, San Diego, CA) as described previously [16], [17]. Briefly, 1×10^5^ alloantigen-specific CTLs were co-cultured with or without 1×10^5^ KI-LCL cells in 0.2 ml of RPMI 1640 medium supplemented with 10% FCS, and then FITC-conjugated anti-CD107a antibody was added to each well. After 3 hours, incubated cells were collected and analyzed by flow cytometry using PE-conjugated anti-CD8 antibody (BD Biosciences, Franklin Lakes, NJ). For analysis of degranulation, the relative log fluorescence of live cells was measured using a FACS flow cytometer (BD Biosciences).

The immunofluorescence intensities of CTLs cultured with and without alloantigen stimulation were measured, and the mean fluorescence index (MFI) was calculated as (mean value for stimulated sample – mean value for non-stimulated sample)/mean value for non-stimulated sample.

## Results

### Genetic subtypes of FHL patients

Among the 31 patients with FHL, 17 appeared to have *PRF1* mutation and lacked expression of perforin protein as measured by flow cytometry and Western blotting, whereas 10 patients appeared to have *UNC13D* mutation and lacked Munc13-4 protein expression as measured by Western blotting. No *STX11* mutations were detected in any of the patients (Table 1). Most of the data have been reported elsewhere [11], [12], [14], [21], [22], [26]. For the remaining 4 patients (UPN28-31), *STXBP2* mutation and CTL function were further analyzed.

**Table 1:** Genetic mutations of *PRF1*, *UNC13D*, *STX11*, and *STXBP2* identified in 31 patients.

UPN	Age/Sex	*PRF1*	*UNC13D*	*STX11*	*STXBP2*
1	3 mo/F	1090.91delCT/1090.91delCT	-	-	-
2	2 mo/F	1090.91delCT/207delC	-	-	-
3	1 mo/F	1090.91delCT/207delC	-	-	-
4	11 y/F	949G>A (M)/1A>G (N)	-	-	-
5	1 mo/F	1083delG/1491T>A (N)	-	-	-
6	4 mo/F	1289insG/1289insG	-	-	-
7	1 mo/F	1349C>T (M)/1349C>T	-	-	-
8	2 mo/F	1090.91delCT/1246C>T (N)	-	-	-
9	12 y/F	1090.91delCT/1228C>T (M)	-	-	-
10	7 y/F	1349C>T (M)/1349C>T	-	-	-
11	2 mo/M	207delC/1122G>A (M)	-	-	-
12	1 mo/M	1090.91delCT/NT	-	-	-
13	4 mo/F	757G>A (M), 253G>A (M)/853-855delAAG	-	-	-
14	1 mo/F	160C>T (M), 272C>T (M)/853-855delAAG	-	-	-
15	3 mo/F	853-855delAAG/1491T>A (N)	-	-	-
16	5 mo/M	1090-1091delCT/1168C>T (N)	-	-	-
17	1 y/M	1090-1091delCT/1349C>T (M)	-	-	-
18	1 mo/M	-	640C>T (M)/-	-	-
19	6 mo/F	-	1596+1g>c (S)/1596+1g>c (S)	-	-
20	4 mo/F	-	766C>T (M)/1545-2a>g (S)	-	-
21	2 mo/M	-	640C>T (M)/1596+1g>c (S)	-	-
22	5 mo/M	-	1596+1g>c (S)/1723insA	-	-
23	5 mo/M	-	1596+1g>c (S)/754-1g>c (S)	-	-
24	6 mo/M	-	754-1g>c (S)/754-1g>c (S)	-	-
25	11 mo/M	-	1596+1g>c (S)/322-1g>a (S)	-	-
26	1 mo/M	-	754-1g>c (S)/2163G>A (N)	-	-
27	2 mo/F	-	322-1g>a (S)/754-1g>c (S)	-	-
28	2 mo/M	-	-	-	292-294delGCG/88-1g>a
29	2 mo/M	-	-	-	1243-1246delAGTG/1243-1246delAGTG
30	0 day/M	-	-	-	-
31	0 day/F	-	-	-	-

UPN, unique patient number; M, male; F, female; -, not detected, NT, not tested.

In parenthesis, M means missense mutation, N means nonsense mutation, and S means splicing abnormality.

### *STXBP2* analysis and Munc18-2 expression in 4 patients with non-FHL2/3/4

**Figure 1:**
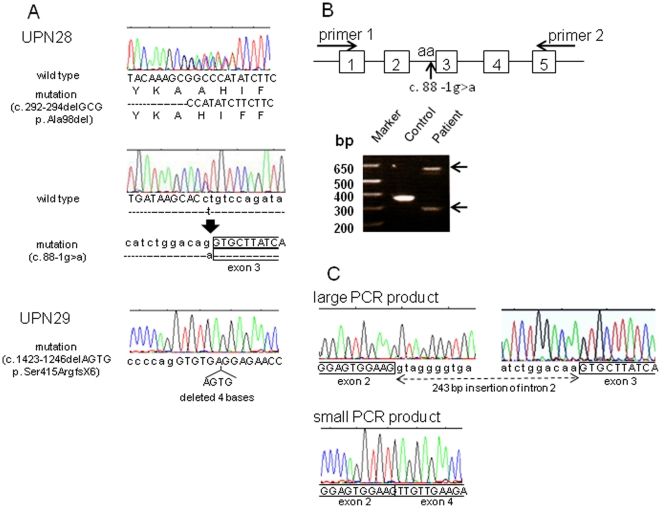
Identification of *STXBP2* mutations. (A) Sequencing analysis of 4 patients with non-FHL2/3/4 and detection of 3 novel mutations in 2 of them: a compound heterozygous mutation of 292_294delGCG resulting in Ala98del at exon 5 (upper panel) and 88-1g>a in intron 2 (lower panel) in one patient (UPN28), and a homozygous mutation of 1243-1246AGTG resulting in Ser415ArgfsX6 at exon 15 in the other (UPN29). (B) Expression of *STXBP2* cDNA in UPN28 with 88-1g>a mutation. Schematic representation of positions of the primers for RT-PCR and 88-1g>a mutation is shown in the upper panel, and for RT-PCR products from 88-1G>A mutation of *STXBP2* in the lower panel. The expected 350-bp product of *STXBP2* exons 1–5 was detected in a healthy control individual, whereas extra larger- and smaller-sized products were detected in UPN28 (arrow). (C) Sequence analysis revealed that the 88-1g>a mutation retained the entire intron 2 (243 bp) in the cDNA. This insertion is predicted to cause addition of 81 amino acids to the N-terminal region of the large Sec1 domain of the Munc18-2 protein (upper panel). Sequence analysis of the smaller fragment revealed that the mutation caused skipping of exon 3 (82 bp), resulting in a frame shift and translational arrest after an additional 20 amino acids (lower panel).

Genetic analysis of *STXBP2* was performed in 4 patients with non-FHL2/3/4 (UPN28-31). As shown in Fig. 1A, a compound heterozygous *STXBP2* mutation with 292_294delGCG and 88-1g>a was detected in UPN28, and a homozygous mutation with 1243_1246delAGTG appeared to be present in UPN29. These 3 mutations of *STXBP2* are all novel. RT-PCR analysis showed that 2 aberrant cDNAs were produced in UPN28 (Fig. 1B). Sequence analysis revealed that the large fragment 88-1g>a mutation caused insertion of the entire intron 2 (243 bp) into the cDNA, while in the small fragment the mutation caused skipping of exon 3 (82 bp), resulting in a frame shift and translational arrest after an additional 20 amino acids (Fig. 1C).

**Figure 2:**
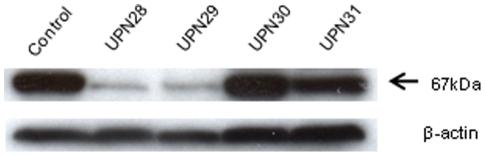
Western blot analysis of Munc18-2 protein expression. Expression of Munc18-2 protein in each CD8^+^ T-cell line that had been stimulated with allogeneic B-LCL cells was analyzed by Western blotting using anti-Munc18-2 antibody. Munc18-2 protein was abundantly detected at 67 kDa in CTL lines established from healthy control individuals and 2 non-FHL2/3/4/5 patients (UPN30, and UPN31).

We analyzed the expression of Munc18-2 protein in CTLs of these 4 patients using Western blotting. As shown in Fig. 2, the Munc18-2 protein band at approximately 67 kDa was scarcely detectable in 2 FHL patients with *STXBP2* mutation (UPN28, UPN29). On the basis of these data, these 2 were diagnosed as having FHL5. On the other hand, Munc18-2 protein expression was clearly detected in CTL lines established from the remaining 2 patients (UPN30, UPN31); therefore, these patients were considered to have FHL with unknown genetic mutations.

### Functional analysis of CTL lines established from FHL patients

**Figure 3:**
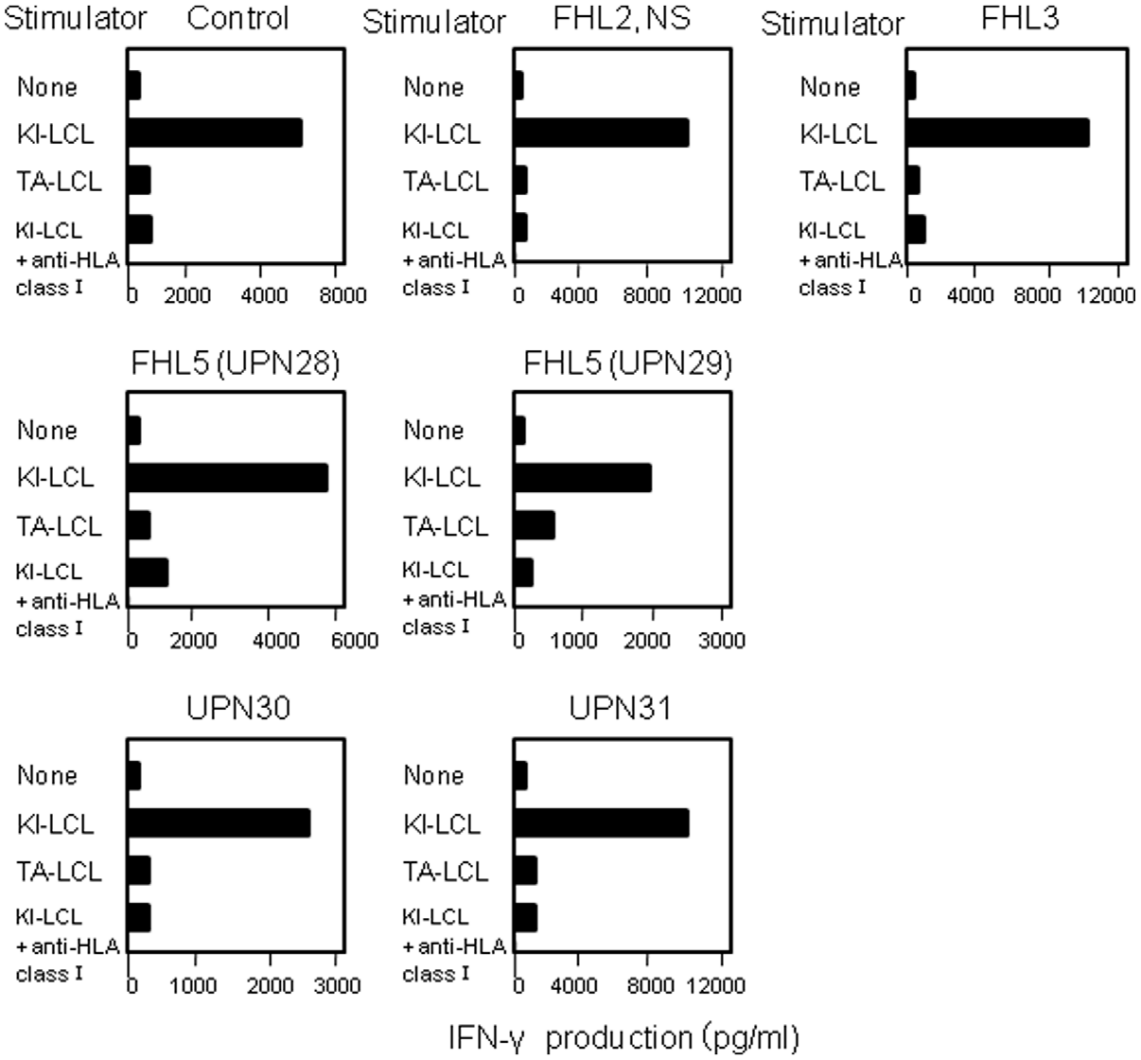
IFN-γ production by alloantigen-specific CD8^+^ T cell lines. CD8^+^ T-cell lines were generated from the PBMCs of the patients with FHL and healthy individuals as controls by stimulation with allogeneic B-LCL (KI-LCL) cells. Responder cells were co-cultured with or without KI-LCL or TA-LCL, which shared no HLA antigens with KI-LCL, in the presence or absence of anti-HLA class I monoclonal antibody for 24 hours. IFN-γ production was measured by ELISA. All FHL patients showed normal production of IFN-γ. The HLA type of KI-LCL is HLA-A01/30, B13/17, Cw6/-, DRB1*0701/*0701, and that of TA-LCL is HLA-A24/26, B62/-, Cw4/w9, DRB1*0405/*0901. NS indicates *PRF1* nonsense mutation.

Alloantigen-specific CD8^+^ CTL lines were generated from healthy individuals, and from patients with FHL2 (UPN8), FHL3 (UPN23), and non-FHL2/3/4 (UPN28-31). The antigen specificities of the T-cell lines were examined by measuring their IFN-γ production in response to stimulation with allogeneic LCL cells. As shown in Fig. 3, all alloantigen-specific CD8^+^ T-cell lines generated by stimulation with allogeneic KI-LCL produced large amounts of IFN-γ in response to stimulation with KI-LCL cells, but not with TA-LCL cells, which share no HLA antigens with KI-LCL. These results indicated that T lymphocytes of FHL patients can respond normally to antigen stimulation and produce inflammatory cytokines. Their IFN-γ production was significantly abrogated by anti-HLA class I antibody, indicating that the responses of these T-cell lines were alloantigen-specific and HLA class I-restricted.

**Figure 4:**
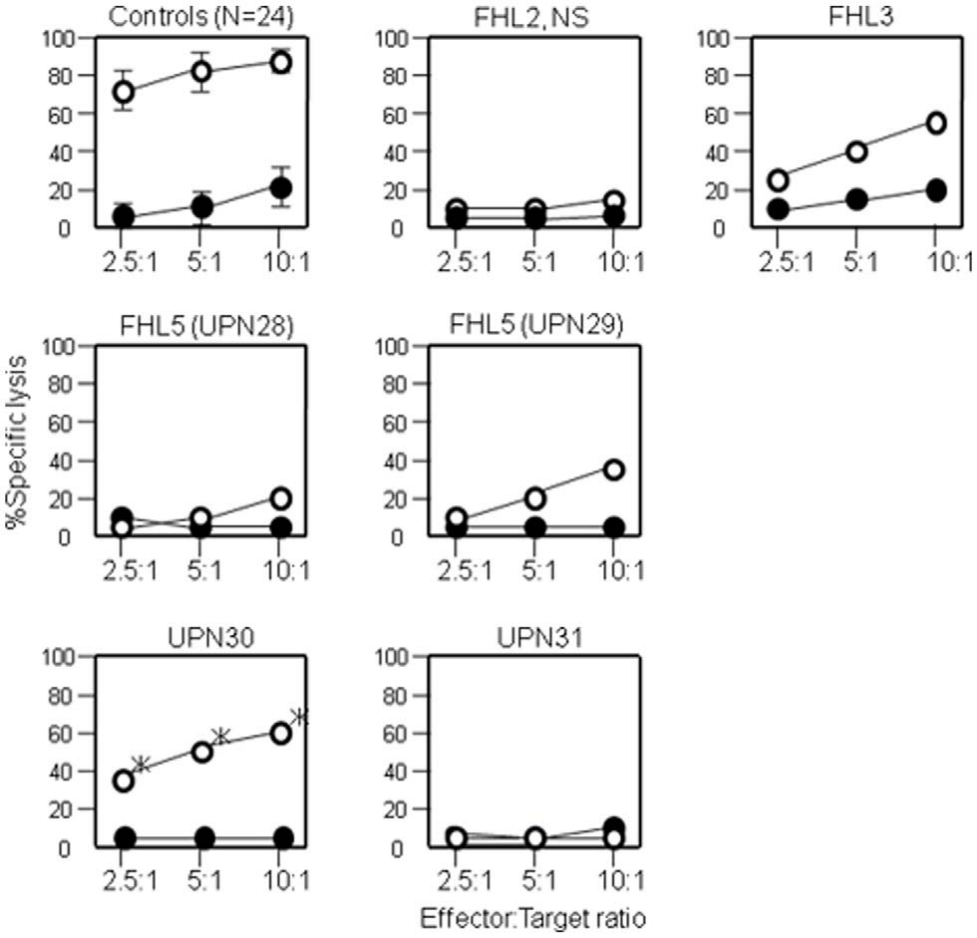
Cytotoxicity of alloantigen-specific CD8^+^ T-cell lines. CD8^+^ T-cell lines were generated from the PBMCs of the patients with FHL and 24 healthy individuals as controls by stimulation with allogeneic B-LCL (KI-LCL) cells. Their cytotoxicity was determined against allogeneic KI-LCL (clear circles) and against allogeneic TA-LCL (solid circles). All FHL patients showed various degrees of impairment of CTL-mediated cytotoxicity against allogeneic B-LCLs. NS indicates *PRF1* nonsense mutation.

Cytotoxic activity mediated by CD8^+^ alloantigen-specific T-cell lines generated from healthy individuals (n = 24) and FHL patients are measured, and the representative data are shown in Fig. 4. Antigen-specific cytotoxicity mediated by CTLs from FHL2 patients with *PRF1* nonsense mutation was entirely deficient, whereas that of CTLs from FHL3 patients with *UNC13D* splicing abnormality was low but still detectable, as we have reported previously [14], [21]. Cytotoxicity mediated by CTLs generated from 2 FHL5 patients also appeared to be low but still detectable. However, the cytotoxicity from 2 patients with unknown genetic mutations was variable; moderately impaired in one (UPN30), and deficient in the other (UPN31).

### Degranulation analysis of CTL lines established from FHL patients

**Figure 5:**
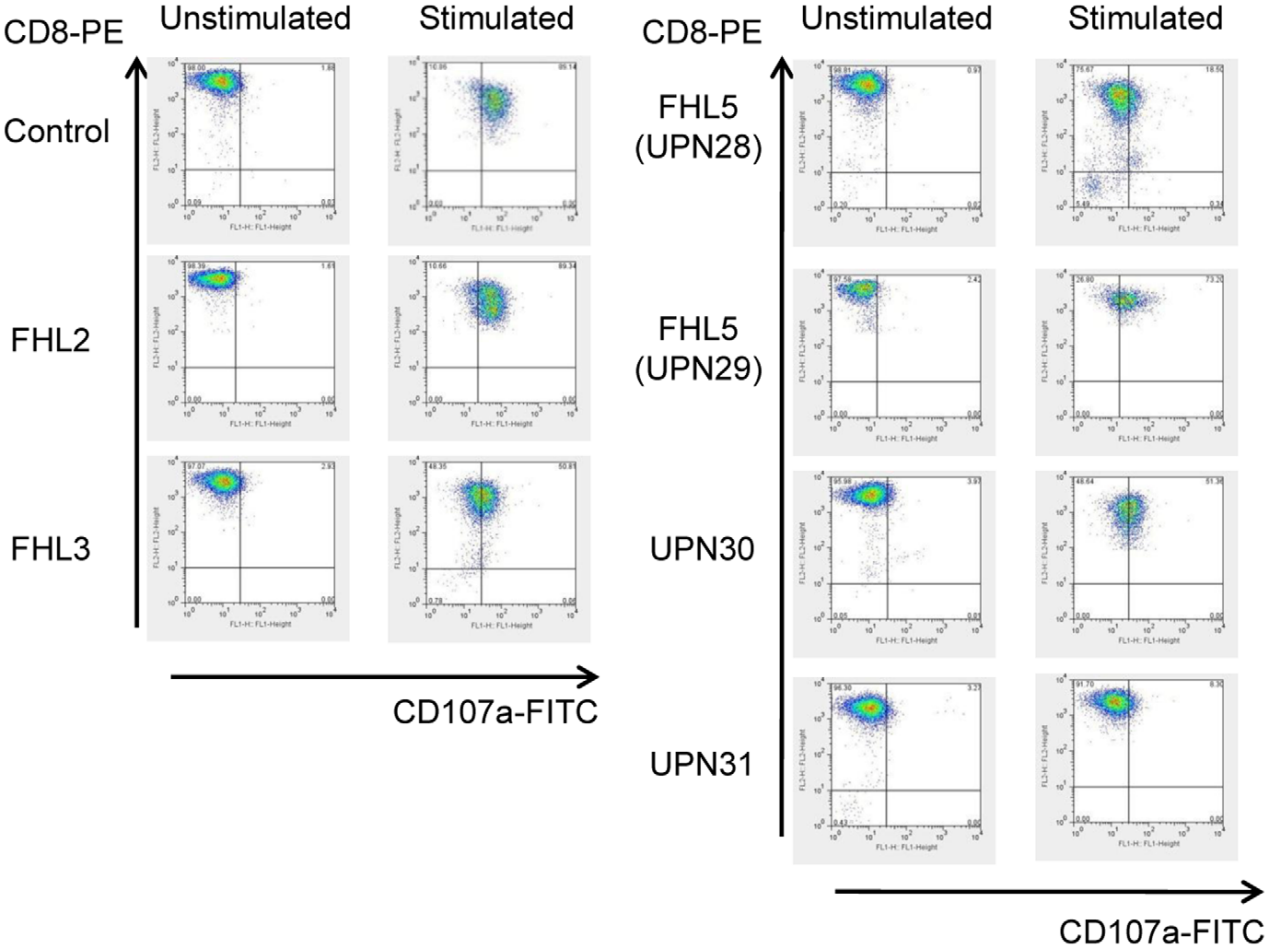
CD107a expression of alloantigen-specific CD8^+^ T-cell lines. Flow cytometric analysis of CD107a expression was performed using CD8^+^ T-cell lines generated from a healthy individual and FHL patients, as detailed in the text. Left panel of each column shows CD107a expression in CD8^+^ T cells without any stimulation. Right panel of each column shows CD107a expression in CD8^+^ T cells stimulated with KI-LCL cells.

Degranulation activity mediated by CTLs established from healthy individuals and FHL patients are measured, and the representative data are shown in Fig. 5. The fluorescence intensities of CTLs cultured with and without alloantigen stimulation were compared by calculating MFI. Both control CTLs generated from healthy individuals and perforin-deficient (FHL2) CTLs showed a marked increase of fluorescence intensity following alloantigen stimulation, indicating that CTLs with perforin deficiency had no impairment of degranulation activity; MFI of CTLs generated from healthy individuals (n = 4) and the patient with perforin deficiency was 4.19±1.15 (mean ± SD) and 5.90, respectively. On the other hand, the increase of fluorescence intensity in Munc13-4-deficient (FHL3) CTLs following alloantigen stimulation was relatively slight; i.e. MFI was 1.81. In repeated experiments, similar degrees of degranulation were detected using CTLs established from other FHL2 or FHL3 patients. CTLs established from 2 FHL5 patients also showed a slight but significant change in fluorescence intensity (MFIs was 1.35). Notably, the increase of fluorescence intensity by CTLs established from 2 patients with unknown genetic mutations was also variable; a slight but significant change in UPN30 (MFI was 1.53), while completely undetectable even after alloantigen stimulation in UPN31 (MFI was 0.16).

### Clinical and laboratory findings of 2 FHL patients with unknown genetic mutations

Clinical and laboratory findings of 2 FHL patients with unknown genetic mutations were analyzed. Both had splenomegaly, deficient NK cell activity and hemophagocytosis in bone marrow, and had shown onset of the disease at birth. One patient (UPN30) also showed hydrocephalus as CNS involvement. They had a positive family history of HLH, i.e. their sibling had shown severe hemophagocytosis and died in infancy. Both received immunochemotherapy with or without stem cell transplantation, but three subsequently died due to disease progression or complications related to the treatment.

## Discussion

We have been performing a continuous nationwide survey of HLH in Japan [27]. Among 87 young patients with HLH registered so far, 31 were diagnosed as having FHL. Among these 31 patients, 17 and 10 patients appeared to have FHL2 and FHL3, respectively, while no FHL4 patient was detected. In the present study, we carried out precise genetic characterizations of 4 non-FHL2/3/4 patients. Among these patients, 2 showed *STXBP2* mutations and were diagnosed as having FHL5. These findings demonstrate that the actual incidence of FHL2 and FHL3 in Japan is approximately 55% and 32%, respectively. FHL5 with *STXBP2* mutation accounts for only 6%, and no FHL4 patients have yet been found in Japan. Since more than 80% of FHL patients in Japan have been registered by our laboratory, these findings reflect the actual epidemiology of FHL in Japan. In a cohort study using samples from West Asian countries, mutations of 3 known genes (*PRF1*, *UNC13D*, *STX11*) were identified in 80% of FHL patients, while *STXPB2* mutation accounted for 10% and the causes remained unknown for the remaining 10% of FHL cases [17]. These data suggest the presence of other gene deficiencies responsible for FHL in various ethnic groups.

*STXBP2* is a newly identified causative gene for FHL5. zur Stadt et al. reported 12 patients with 9 kinds of *STXBP2* mutations from Turkey, Saudi Arabia, and Central Europe [16]. Cote et al. also reported 9 patients from Turkey, Saudi Arabia and Palestine [17]. Among *STXBP2* mutations in FHL5, 1430C>T resulting in P477L and 1247-1g>c resulting in a splicing effect are the most frequent mutations in these countries [16], [17]. The association between phenotype and genotype in FHL5 is still obscure. The former report described that patients with mildly impaired CD107 expression or residual CTL activity showed late onset [16]. The latter report mentioned that most of the FHL5 patients with 1430C>T showed very early onset and rapid death, whereas all of the patients with splice site mutation developed their symptoms several years later [17]. In the present study, 3 novel mutations of *STXBP2* were identified in 2 Japanese patients. Both of these patients showed onset in early infancy and the cytotoxic activities of their CTLs and NK cells were low. Further accumulation of FHL5 patients should make it possible to clarify the relationship between phenotype and genotype in this disease.

Bryceson et al. [28] demonstrated that syntaxin11 deficiency is predominantly manifested in the context of NK, rather than CD8^+^ CTLs. Two recent studies [16], [17] have shown that Munc18-2 deficiency is strongly manifested at the level of naive NK cells, whereas relatively milder defects are evident in CD8^+^ CTLs. These studies suggest that NK deficiency is the likely trigger for at least two types of FHL (FHL4 and FHL5), while perforin and Munc13-4 deficiencies affect both cell types and thus the trigger cannot be discriminated. However, the number and cytotoxic function of NK cells vary depending on a number of factors, including the nature of the disease, infections, and type of treatment, as indicated by Bryceson et al. [28]. Therefore measurements of NK cell activity using whole PBMCs may not accurately reflect the immune status of the patients [21]. We therefore established alloantigen-specific CTL lines from patients with the different subtypes of FHL and compared their cytotoxic activities. Consequently, CTL lines generated from 2 FHL5 patients showed markedly decreased but detectable cytotoxicity with a level similar to that in FHL3. In the SNARE systems, perforin is critical for granzyme delivery, and Munc13-4 is essential for priming of cytotoxic granules docked at the immunologic synapse, whereas syntaxin11 regulates membrane fusion events [29], [30]. Via interaction with syntaxins, Munc18 proteins are required for secretory vesicle docking and fusion with the immunologic synapse [31], [32]. A recent report has indicated that docked vesicles are primed for fusion by Munc13-4 when Munc18-2 clasps across the zippering 4-helix-assembled trans-SNARE complex [33]. These findings suggest that at the immunologic synapse of CTLs, the Munc18-2/syntaxin11 complex could play a role similar to that of Munc13-4 by regulating granule docking and the initiation of SNARE formation prior to the priming step. Our data indicating that the cytotoxic activities of CTLs and NK cells in FHL3 and FHL5 are impaired to a similar degree appear to support this hypothesis.

Interestingly, the degrees of cytotoxic activity mediated by CTL lines generated from 2 patients with unknown genetic mutations appeared to be significantly different, i.e. moderately decreased in one and undetectable in the other, as is the case for *PRF1* nonsense mutation [21]. A large amount of IFN-γ was produced by both of the CTL lines generated from these patients after stimulation with allogeneic LCL cells, and this cytokine production was abrogated by anti-HLA class I antibody, indicating that the antigen-recognition system mediated via the T-cell receptor/CD3 complex was intact in both cases. These data also indicate that immunological synapses are normally formed between CTLs from these FHL patients and target cells.

A recent study has indicated that CD107a expression mediated by antigen stimulation is a good candidate marker for the cytotoxic activity of CTLs and NK cells [34]. The lysosome-associated membrane protein-1, also known as CD107a, is usually located in cytotoxic granules in CTLs and NK cells. During the cytotoxic activity of CTLs and NK cells, these molecules are transported to the cell surface. Therefore, the level of CD107a expression is well correlated with degranulation activity in CTLs and NK cells. Indeed, activated NK cells derived from patients with FHL3 showed a sharply lower frequency and MFI of CD107a staining compared with healthy control subjects [35]. CD107a assay is effective tool for rapid identification of patients with FHL3 and other impaired degranulation. Furthermore, it has been reported previously that degranulation in Munc18-2-deficient CTLs is significantly impaired [16], and that transfection of these cells with the wild-type *STXBP2* gene results in recovery of the degranulation activity [17]. In our present study, determination of CD107a expression by flow cytometry indicated that Munc18-2-deficient CTLs also showed a significantly reduced level of degranulation activity. Similarly to cytotoxic activity, the degree of degranulation mediated by CTL lines generated from 2 patients with unknown genetic mutations appeared to differ significantly. That is, degranulation activity was moderately impaired in one patient and severely impaired in the other. These data also strongly suggest the presence of two types of FHL with unknown genetic mutation.

In summary, we have examined the genetic and immunological abnormalities in Japanese patients with different FHL subtypes, and our data have clarified the frequency of each FHL subtype in Japan, as well as strongly suggesting that unknown FHL subtypes are present. Further investigations to identify the molecular defects in these FHL patients will be required to clarify the pathogenesis of FHL. It is also expected that further progress in the study of FHL may clarify the detailed mechanisms of CTL- and NK cell-mediated cytotoxicity.

## Supporting Information

Table S1 Primer sets for mutation screening of *STXBP2*.
(0.06 MB DOC)
