# Supplementary material for: Subtypes of Familial Hemophagocytic Lymphohistiocytosis in Japan Based on Genetic and Functional Analyses of Cytotoxic T Lymphocytes
Source: PLoS One. 2010 Nov 30;5(11):e14173. doi: 10.1371/journal.pone.0014173 (PMC2994802; doi:10.1371/journal.pone.0014173)
Supplement: Table S1 — Primer sets for mutation screening of STXBP2. (0.06 MB DOC) [file pone.0014173.s001.doc]

| **Table S1. Primer sets for mutation screening of *STXBP2*** | | | | | | | | | | |
| --- | --- | --- | --- | --- | --- | --- | --- | --- | --- | --- |
| Primer name |  | Forward primer sequence |  | Reverse primer sequence |  | Target exon |  | Expected size (bp) |  | Primer for sequencing |
| M18-2-p1 |  | GTGCACCTCCAGCGACTT |  | CGCCATGAAACCCAGGAT |  | 1 |  | 351 |  | forward |
| M18-2-p2 |  | GGACCAGAGAACCAGCATTC |  | CAGAACCCATTCACCCAACT |  | 2 |  | 222 |  | reverse |
| M18-2-p3 |  | CTGCTCCTCCATCCATCTGT |  | GGGAGTTCTGGACGATTTGA |  | 3 |  | 233 |  | reverse |
| M18-2-p4 |  | TCTCCTGCAGTCCCTCCTAA |  | CGCATACACACACGCTCACT |  | 4 |  | 233 |  | forward |
| M18-2-p5 |  | GTTTGCACATGGTGGCAGAT |  | ACCAACACCCTAGGCTCTCC |  | 5 |  | 207 |  | forward |
| M18-2-p6 |  | GACCCCGACTTTCACCTACA |  | GGGACATTTGGTCACAAACC |  | 6 |  | 295 |  | forward |
| M18-2-p7 |  | CTCCAATTCGGCAAAGCAG |  | TAGGTGTTGCACGCGGTTA |  | 7 |  | 340 |  | forward |
| M18-2-p8 |  | GCGTGCAACACCTAACCTTT |  | CCCTCACCTCCCAAGCAC |  | 8 |  | 246 |  | forward |
| M18-2-p9 |  | CAGTCTGGGCGAGGTGAG |  | GACGGGTTCCAAGTCTGC |  | 9 |  | 250 |  | forward |
| M18-2-p10 |  | CTGCTGGACATAGAGCAGGA |  | CGCTACCAGGCCCACAGT |  | 10 |  | 289 |  | forward |
| M18-2-p11 |  | CCCTCGTGTGACTCCAGACT |  | ACCAGCGCCTTTGGTGAC |  | 11 |  | 217 |  | forward |
| M18-2-p12 |  | GGCCACAGCCTGGATTTC |  | AGACAGAGCATGGGGTTGAG |  | 12 |  | 220 |  | forward |
| M18-2-p13 |  | AGCCTTTGTTATCCCCCAAC |  | CCCTCCACCTCTCCACAAG |  | 13 |  | 243 |  | reverse |
| M18-2-p14 |  | AGGACCCAAATGTCCTCTTG |  | GGAGACGCTGGCAAATGG |  | 14 |  | 299 |  | forward |
| M18-2-p15 |  | CCACTGCAAGGTTCTCTCATC |  | CTGTGATCCGCACCCTCT |  | 15 |  | 276 |  | forward |
| M18-2-p16 |  | CTCTGTTCCACTTCCCCATC |  | CCTGACCTGCCACCCAGT |  | 16 |  | 240 |  | forward |
| M18-2-p17 |  | ATGCTGGTTCCTGTCTGC |  | CGCCCACCTAACACAGACC |  | 17 |  | 250 |  | forward |
| M18-2-p18 |  | AGGCCGCTGTCAGGTGAG |  | GTCCCAGTCCTGGTGACTT |  | 18 |  | 298 |  | forward |
| M18-2-p19 |  | AGTCAGGGACCTGGTTTGCT |  | CTCTGGAAAGGGAGGGGTAG |  | 19 |  | 241 |  | forward |
